# Reduction of phase noise in nanowire spin orbit torque oscillators

**DOI:** 10.1038/srep16942

**Published:** 2015-11-23

**Authors:** Liu Yang, Roman Verba, Vasil Tiberkevich, Tobias Schneider, Andrew Smith, Zheng Duan, Brian Youngblood, Kilian Lenz, Jürgen Lindner, Andrei N. Slavin, Ilya N. Krivorotov

**Affiliations:** 1 Department of Physics and Astronomy, University of California, Irvine, CA 92697, USA; 2 Institute of Magnetism, National Academy of Sciences of Ukraine, Kyiv 03142, Ukraine; 3 Department of Physics, Oakland University, Rochester, MI 48309, USA; 4 Helmholtz-Zentrum Dresden - Rossendorf, Institute of Ion Beam Physics and Materials Research, Bautzner Landstraße 400, 01328 Dresden, Germany

## Abstract

Spin torque oscillators (STOs) are compact, tunable sources of microwave radiation that serve as a test bed for studies of nonlinear magnetization dynamics at the nanometer length scale. The spin torque in an STO can be created by spin-orbit interaction, but low spectral purity of the microwave signals generated by spin orbit torque oscillators hinders practical applications of these magnetic nanodevices. Here we demonstrate a method for decreasing the phase noise of spin orbit torque oscillators based on Pt/Ni_80_Fe_20_ nanowires. We experimentally demonstrate that tapering of the nanowire, which serves as the STO active region, significantly decreases the spectral linewidth of the generated signal. We explain the observed linewidth narrowing in the framework of the Ginzburg-Landau auto-oscillator model. The model reveals that spatial non-uniformity of the spin current density in the tapered nanowire geometry hinders the excitation of higher order spin-wave modes, thus stabilizing the single-mode generation regime. This non-uniformity also generates a restoring force acting on the excited self-oscillatory mode, which reduces thermal fluctuations of the mode spatial position along the wire. Both these effects improve the STO spectral purity.

The discovery of giant spin Hall effect in nonmagnetic heavy metals such as Pt^1^,^2^,^3^, W^4^ and Ta^3^,^5^,^6^,^7^ creates new opportunities for the manipulation of magnetization by spin currents, including switching and excitation of self-oscillations of magnetization^4^,^5^,^6^,^8^,^9^,^10^,^11^. Spin-orbit interaction in such heavy metals results in large spin-dependent deflection of electrons participating in electric charge current^12^,^13^,^14^,^15^,^16^, which can be viewed as a pure spin current flowing perpendicular to the charge current^17^,^18^,^19^. This pure spin current can be injected from the heavy metal into an adjacent ferromagnet and apply spin torque to its magnetization^20^,^21^. Due to its non-conservative nature, this spin orbit torque can act as magnetic anti-damping^22^,^23^ leading to the decrease of the relaxation rates of spin waves (SWs), which was observed in both metallic^2^,^24^,^25^,^26^ and insulating^3^,^27^ ferromagnets.

A spin orbit torque uniformly applied to a spatially extended ferromagnetic film cannot reduce the spin wave damping to zero, and therefore, cannot excite self-oscillations of magnetization in the film even at high spin current densities^28^. The origin of this anti-damping saturation is non-linear spin wave interactions, which distribute the injected energy and angular momentum among a continuum of SWs with different wave vectors, so that the net damping rate for any SW mode remains positive^8^. Patterning of the ferromagnetic film into nanoscale dots discretizes the spin wave spectrum and closes many of the nonlinear SW scattering channels. In such a case, the relaxation rate of the lowest-energy SW mode of the nanodot can reach zero, and self-oscillations of the mode can be excited by the spin current^9^. Another route to excitation of self-oscillations by spin orbit torques is the application of a high spin current density to a nanoscale region of an extended ferromagnetic film by using the current concentrators^8^,^10^,^29^,^30^. In this case a nonlinear self-localized spin wave “bullet” mode can be excited^10^,^31^,^32^. Since the frequency of the bullet mode lies below the SW spectrum, the resonant scattering processes from this mode into a SW continuum are forbidden, which enables the self-sustained excitation of this large-amplitude mode^8^,^10^,^29^,^30^. The characteristic dimensions of this bullet mode is determined by the exchange length of the ferromagnet, and are typically below 100 nm^31^,^33^. Recently, we have shown that the spin orbit torques can excite self-oscillations of magnetization in micrometer-scale ferromagnets, namely in quasi-one-dimensional ferromagnetic nanowires^11^. The geometric confinement of the spin waves in nanowires suppresses some nonlinear scattering channels such as four-magnon scattering^34^,^35^, which turns out to be sufficient for the excitation of sustainable self-oscillations over micrometer-scale regions of the nanowire^11^. In spite of the large excitation volume and the associated diminished impact of the random thermal torques on the magnetization dynamics, the spectral linewidth of the microwave signal generated by nanowire STOs was found to be comparable to that of nanoscale STOs^22^,^36^,^37^,^38^,^39^,^40^,^41^,^42^,^43^,^44^,^45^,^46^,^47^,^48^,^49^,^50^. This result can be attributed to the simultaneous excitation of several SW modes in nanowire auto-oscillators, because it is known that the interactions between the simultaneously excited self-oscillatory modes can substantially increase the linewidths of the generated modes^51^,^52^. Therefore, new methods for selective excitation of a single self-oscillatory mode in a nanowire STO are highly desirable for the development of STO devices with high spectral purity. In this paper, we report experiments demonstrating that the single-mode regime of operation and the associated phase noise reduction can be achieved via proper design of the nanowire STO shape. We show that STOs based on tapered nanowires, such as the one shown in Fig. 1(b,d) exhibit reduced phase noise, and a wider bias current range of single mode operation in comparison to the straight nanowire STOs of similar dimensions. We employ numerical simulations to show that the spatial non-uniformity of the spin current density in the tapered nanowire STO is the key factor contributing to the improved phase noise. These simulations also reveal that the spin orbit torques excite a self-localized micrometer-scale bullet mode if nanowire SW modes exhibit negative nonlinear frequency shift. In the tapered nanowire devices, the spatial nonuniformity of the spin current density stabilizes the single-mode generation regime at higher bias currents. It also generates a confining potential for the bullet, which reduces the thermal fluctuations of the spatial position of this mode along the nanowire length, resulting in the reduction of the STO phase noise.

## Results

### Experiment

**Figure 1:**
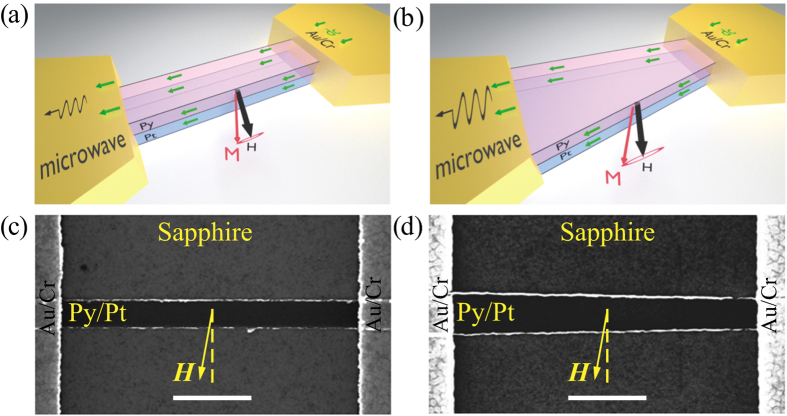
Samples. Schematic of the straight (**a**) and tapered (**b**) nanowire STO: applied magnetic field, electric bias current and precessing magnetization are shown by black, green and red arrows, respectively. Scanning electron micrographs (SEM) of the straight (**c**) and tapered (**d**) nanowire STO samples. 500 nm white scale bars are shown in each SEM image.

The nanowire STO samples based on AlO_*x*_(2 nm)/Py(5 nm)/Pt(7 nm) multilayers were patterned on a sapphire substrate via e-beam lithography and liftoff as described in Methods (here Py = Permalloy = Ni_80_Fe_20_). The wires are 6 *μ*m long with two Au(35 nm)/Cr(7 nm) leads attached to each wire as shown in Fig. 1. The 1.9 *μ*m long nanowire section between the leads is the STO active region, in which direct bias current applied to the wire generates the anti-damping spin orbit torque. The width of the straight nanowires is 190 nm, while the width of the tapered nanowires increases from 190 nm to 250 nm within the active region.

In our measurements, the magnetization of nanowires is saturated by a 700 Oe in-plane external magnetic field applied at the angle of 80° with respect to the wire axis as shown in Fig. 1. Direct current *I*_*dc*_ applied to the nanowire excites self-oscillations of magnetization when the bias current exceeds a certain critical value (*I*_*dc*_ > 4.45 mA for the straight wire and *I*_*dc*_ > 5.675 mA for the tapered wire). The critical current is higher for the tapered nanowire because of its greater average width. Magnetization self-oscillations are converted into a microwave signal via anisotropic magneto-resistance of the Py layer^11^. The output microwave signal is amplified by a low-noise amplifier and measured by a spectrum analyzer. All measurements reported in this paper were made at the bath temperature of 4.2 K, although the sample temperature near the critical current is approximately 150 K due to heating of the nanowire by the bias current^11^. We studied 3 straight and 3 tapered nanowire samples and found similar results for all these devices. In this paper, we present the data for one representative straight and one representative tapered nanowire STO.

**Figure 2:**
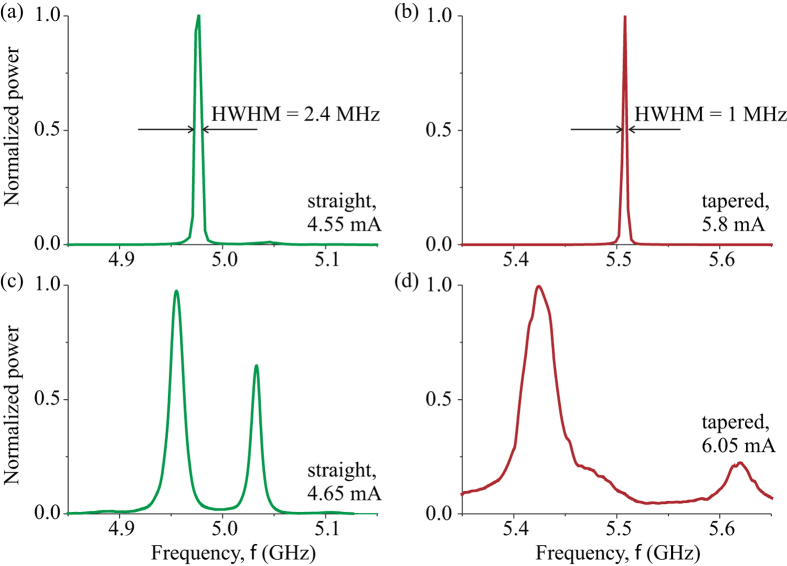
Microwave emission spectra. Normalized power spectra generated by the straight (**a**,**c**) and tapered (**b**,**d**) nanowire STOs at two bias current values.

The spectra of microwave signals generated by the straight and tapered nanowire STOs at two bias current values above the critical currents are presented in Fig. 2. Here we only show the low-frequency group of peaks observed in the spectra. At higher bias currents, we have also detected a group of low-amplitude peaks at 0.8 GHz above the low frequency group. Following the analysis of Ref. 11, we identify the low-frequency peaks as the spin wave modes localized at the wire edges (“edge modes”)^53^,^54^, while the high-frequency peaks arise from the spin wave modes that have their maximum amplitudes within the central part of the wire (“bulk modes”). We also directly verified these conclusions using micromagnetic simulations of the spin wave spectra in the straight and tapered nanowires, as discussed in the next section. Since the critical current for the excitation of bulk modes is significantly higher than the range of the bias currents discussed in this work, we do not discuss the bulk modes in the rest of this paper.

**Figure 3:**
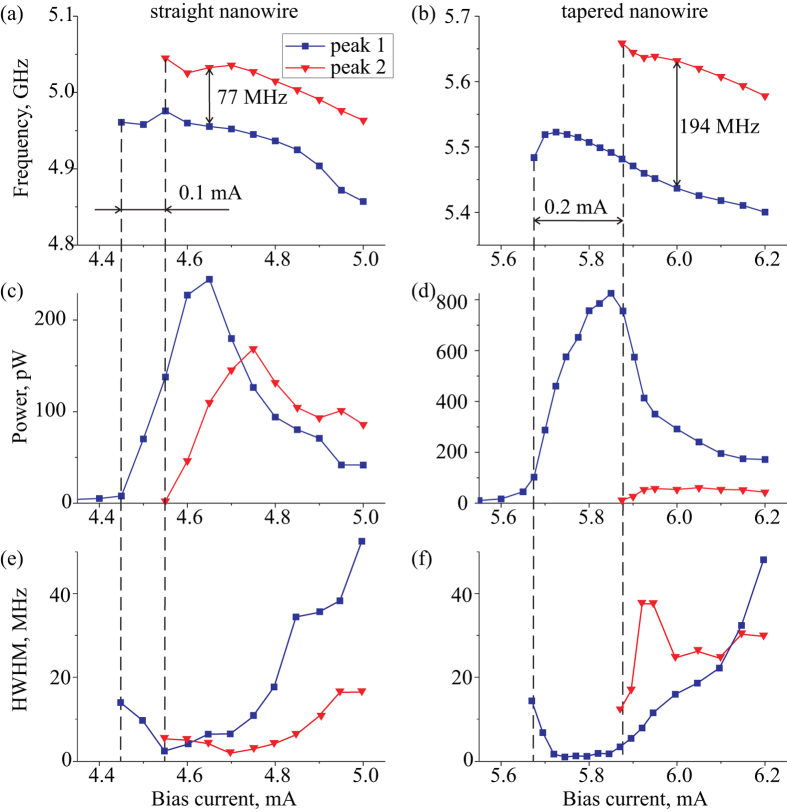
Auto-oscillatory mode frequency and power. Measured dependence of the auto-oscillation frequency (**a**,**b**), integrated microwave emission power (**c**,**d**) and HWHM (**e**,**f**) on the bias current for the first (blue squares) and second (red triangles) peaks in the auto-oscillation spectra.

For both the straight and tapered nanowire STOs, we observed a single spectral peak in a range of bias currents above the critical current *I*_*c*_ (Fig. 2(a,b)). The frequency of this first peak is higher in the tapered nanowire STO than in the straight nanowire STO. This happens because the average demagnetizing field decreases with the increase of the wire width and the average tapered wire width is greater than that of the straight wire. The emission power in the first peak increases with increasing bias current until the second peak appears in the spectrum at the second critical current (*I*_2_ = 4.55 mA for the straight nanowire STO; *I*_2_ = 5.875 mA for the tapered nanowire STO) as shown in Fig. 2(c,d). For *I* > *I*_2_, the integrated power in the first mode decreases, and the spectral linewidth of this mode increases with increasing current, as illustrated in Fig. 2(c,d) and 3. At even higher bias current values, the third edge mode appears in the microwave emission spectra. Here we will not discuss this complicated regime, and will restrict our discussion to the single- and double-mode STO operation regimes, focusing on the differences between the straight and tapered nanowire devices.

While the general features of the emission spectra as a function of the bias current are qualitatively similar for the straight and tapered nanowire STOs, there are significant quantitative differences. First, the current range of the single-mode operation of the tapered nanowire STO (0.2 mA) is twice as wide as that for the straight nanowire STO (0.1 mA) as illustrated in Fig. 3. Second, the frequency gap between the first and the second peaks in the generation spectrum is much wider for the tapered nanowire STO in comparison to the straight nanowire STO. Third, the minimum spectral linewidth (half width at half maximum or HWHM) in the single-mode auto-oscillation regime is significantly smaller for the tapered nanowire STO (1.0 MHz) than for the straight nanowire STO (2.4 MHz), as shown in Fig. 2. All these large quantitative differences between the two types of the STO cannot be attributed to a small difference of the average demagnetization fields, which results in a 10% difference in the edge mode frequency. In the next section we explain the origin of the observed significant impact of the nanowire shape on the spectral properties of nanowire STOs.

### Theory

**Figure 4:**
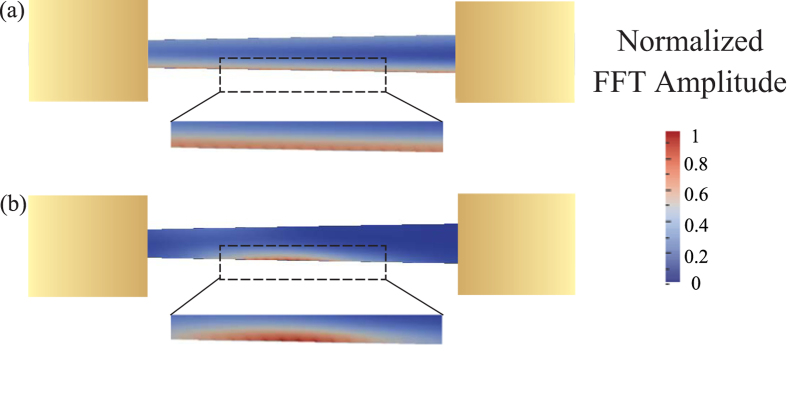
Micromagnetic simulations. (**a**) Spatial profile of the lowest-frequency linear SW mode of the tapered nanowire. (**b**) Spatial profile of the self-oscillatory bullet mode excited by direct current exceeding the critical value. Dashed rectangles show a zoomed in view of the edge mode. Yellow rectangles represent the Au/Cr leads.

In order to visualize the spatial profiles of the observed self-oscillatory edge modes, we performed micromagnetic simulations of the magnetization dynamics in the nanowire STO. First, we employed the spectral mapping technique to calculate the edge spin wave mode profile in the linear regime (see Methods for details). Since the applied magnetic field makes different angles to the two edges of the tapered nanowire sample, the frequencies of the modes localized at the opposite edges are different. The spatial profile of the lowest-frequency edge mode calculated by the spectral mapping technique for the tapered nanowire STO is shown in Fig. 4(a). The mode extends over the entire nanowire length and is strongly localized near one of the edges. We have also performed micromagnetic simulations of the self-oscillatory magnetic dynamics in this nanowire sample driven by spin orbit torque. Such simulations are very time-consuming because of the significant spatial extent of the nanowire and long transient dynamics. For this reason, we performed these simulations for only a few values of the bias current above the critical current. The spatial profile of the self-oscillatory mode driven by direct current is shown in Fig. 4(b). It is clear from this figure that the self-oscillatory nonlinear mode directly originates from the linear edge mode but shows a higher degree of localization near the middle of the nanowire STO active region. We also applied this type of micromagnetic analysis to the straight nanowire STO and observed a similar behavior with the main difference being localization of the edge mode at both edges of the nanowire due to the higher symmetry of the system. We note that in real nanowire samples, equivalence of the two edges is broken due to the random edge roughness and non-uniform edge damage, and thus the localization of the lowest-frequency edge mode near one of the edges is expected as well.

Since detailed micromagnetic simulations of the self-oscillatory dynamics as a function of the direct bias current are prohibitively time-consuming, we developed a one-dimensional model describing current-driven magnetization dynamics in a nanowire STO. This model describes magnetization dynamics in the framework of nonlinear Ginzburg-Landau equation that is derived as a small-amplitude approximation of the Landau-Lifshitz equation:


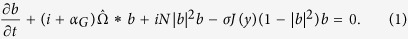


Here *b* = *b*(*y*, *t*) is the complex amplitude of the dynamic magnetization in the excited SW mode, which depends only on the coordinate along the wire axis (*y*-axis), *α*_*G*_ is the Gilbert damping constant, *N* is the nonlinear frequency shift, *J*(*y*) is the spatial distribution of the bias current density along the nanowire axis and *σ* is the spin orbit torque efficiency constant (see Methods). The one-dimensional model is valid if one can factorize the two-dimensional mode profile as *b*(*x*, *y*) = *b*(*y*)*f*(*x*), where *f*(*x*) does not depend on *y*. We explicitly verified that this assumption is valid via micromagnetic simulations of the mode profile shown in Fig. 4.

The frequency operator 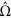 is given by:


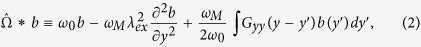


where *ω*_0_ is the spin wave resonance frequency in the linear regime, *ω*_*M*_ = *γμ*_0_*M*_*s*_ and *λ*_*ex*_ is the exchange length. In contrast to the previous studies^31^, we also take into account the magnetodipolar interaction in the Damon-Eshbach geometry via the magnetostatic Green’s function *G*_*yy*_. In all simulations presented in this paper, we neglect the current-induced Oersted field (<50 Oe), which is much smaller than the applied field of 700 Oe. We verified the inclusion of the Oersted field into our Ginzburg-Landau simulations does not significantly change the simulation results.

The sign of the nonlinear frequency shift *N* is the key factor determining the type of self-oscillatory magnetization dynamics driven by spin orbit torque. It is known that for negative nonlinear shift, a nonlinear self-localized solitonic bullet mode is favored under the action of anti-damping spin torque; while for positive nonlinear shift, no self-localization is found^10^,^31^,^32^. Figure 3 demonstrates that the nonlinear frequency shift of the edge SW mode in our system is *negative* (*N* < 0), and, therefore, we should expect self-localization of the excited self-oscillatory mode. We also note that in the previously studied nanowire STO samples prepared via Ar plasma etching^11^, positive nonlinear shift of the edge mode was observed, and the nonlinear mode self-localization did not take place. This demonstrates a strong sensitivity of the nanowire STO properties to the degree of magnetic edge damage, which depends on the sample fabrication technique (see Methods for details).

Figure 4(b) illustrates that the excited self-oscillatory mode given by micromagnetic simulations is indeed a nonlinear self-localized bullet mode, as expected for *N* < 0. The self-localization is evident from the smaller spatial extent of the nonlinear mode in Fig. 4(b) along the wire length compared to the size of the linear mode in Fig. 4(a). The characteristic dimension of this bullet mode is approximately 1 *μ*m, which is one order of magnitude larger than the size of the self-oscillatory bullet modes excited by spin torque in extended thin films in point contact STOs^10^,^31^,^32^,^33^,^40^,^42^. Such increase of the bullet size is a result of the enhanced role of the magnetic dipole interaction in the nanowire geometry. A detailed study of the effect of dipolar interaction on the bullet size will be presented elsewhere.

The frequency of the self-oscillatory mode at the critical current given by micromagnetic simulation (5.8 GHz) is similar to the measured frequency of 5.5 GHz. The value of *N* derived from our micromagnetic simulations is approximately one third of the measured value. This is not surprising because we find N to be very sensitive to the wire fabrication process, and the value of *N* calculated for a nanowire with ideal edges can significantly differ from the measured value of *N*.

**Figure 5:**
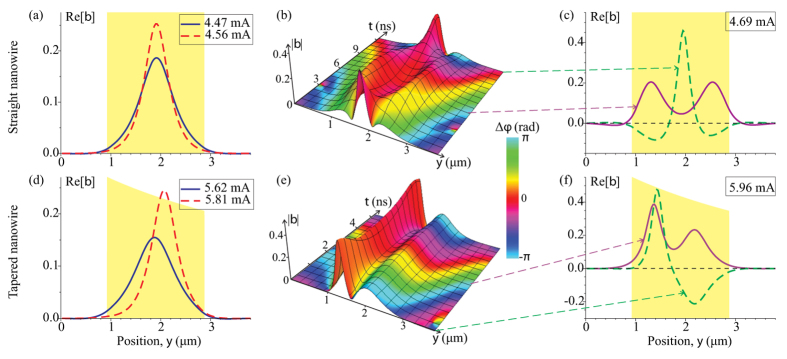
Self-oscillatory mode profiles. Profiles of the self-oscillatory bullet mode in the single-mode regime calculated from Eq. (1) for the straight (**a**) and tapered (**d**) nanowire STOs at different bias currents. The height of the yellow shaded area represents the spin current density within the STO active region. (**b**,**e**) Time evolution of the amplitude 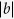 and relative phase Δ*ϕ* (see Methods) of the magnetization oscillations in the double-bullet regime of self-oscillations. (**c**,**f**) Snapshots of the dynamic magnetization profile Re[*b*] at two values of time.

Figure 5 illustrates the bullet mode profiles obtained from the numerical solution of the Ginzburg-Landau equation (see Methods for details). In the straight nanowire STO, a single bullet mode with its maximum in the center of the wire is excited above the critical current as shown in Fig. 5(a). The amplitude of this bullet mode increases and its width decreases with increasing bias current, which is a clear evidence of the nonlinear self-localization. When the current density reaches a second critical current *I*_2_, another bullet mode is excited within the STO active region as illustrated in Fig. 5(c). A similar type of the two-bullet excitation was previously observed in a point contact STO^55^. The double-bullet solution at *I* > *I*_2_ is not stationary – the magnetization profile oscillates between the single-bullet and double-bullet configurations, as illustrated in Fig. 5(b,c). As a result of these mode profile oscillations, the spectrum of the voltage signal generated by the STO develops two prominent peaks in agreement with our experimental observations. At higher currents, additional bullet modes sequentially enter the active region, and the resulting magnetization dynamics becomes very complex.

For the tapered nanowire STO, we solve the same Ginzburg-Landau equation, but with a spatially dependent current density *J*(*y*) ~ 1/*w*(*y*), where *w*(*y*) is the nanowire width. Similar to the straight nanowire case, a nonlinear edge bullet mode is excited at the threshold current *I*_*c*_. With increasing bias current, the size of this bullet mode decreases and the center of the mode shifts towards the wider end of the nanowire as shown in Fig. 5(d). At a higher critical current *I*_2_, a second bullet mode appears in the left part of the active region (where current density is higher). In contrast to the straight nanowire, where the mode profile oscillates between one- and two-bullet mode configurations, two different bullet modes coexist at all times in the tapered nanowire, as evident from Fig. 5(e). These two bullets oscillate with different frequencies – the left bullet experiencing higher current density has a lower oscillation frequency, as illustrated in Fig. 5(e) by the linear increase of the phase difference between the two bullet solutions Δ*ϕ* with time (see Methods for details). This can be also seen in Fig. 5(f) where the two different bullet modes are in phase at one moment of time, and have opposite phases at a later moment. The Fourier transform of the voltage signal arising from this double-bullet dynamics exhibits two distinct spectral peaks corresponding to the two different bullet mode frequencies.

**Figure 6:**
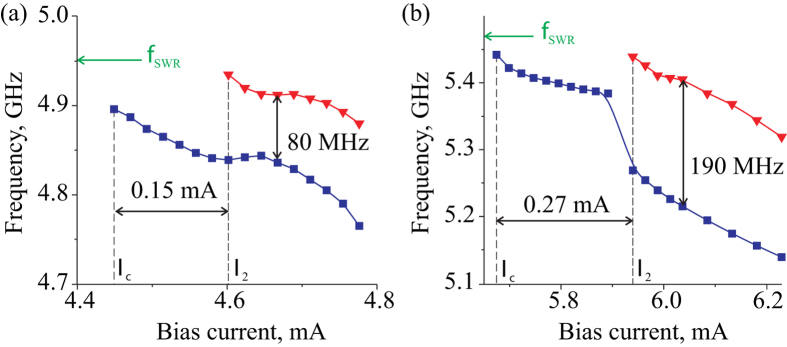
Frequency versus bias current: simulations. Self-oscillatory mode frequency as a function of the bias current calculated by numerically solving Eq. (1) for the straight (**a**) and tapered (**b**) nanowire STOs; blue squares - first bullet mode, red triangles - second bullet mode. Lines are guide for eyes and green arrows show the resonance frequency of the edge mode in the linear regime *f*_SWR_ = *ω*_0_/2*π*.

Figure 6 shows the calculated self-oscillatory mode frequencies given by Eq. (1) versus the magnitude of the direct bias current. These dependences are in good agreement with the experimental data in Fig. 3: the single-mode current range (*I*_2_ − *I*_*c*_) and the inter-mode frequency gap in the double-mode regime are significantly wider for the tapered nanowire STO. The wider single-mode current range is a direct consequence of the current-induced shift of the position of the first bullet mode away from the center of the active region towards the wider end of the nanowire (towards lower current density). This current-induced shift allows the first bullet mode to remain within a region of lower current density (and thus, within a single-mode regime) over a wider range of the applied bias currents. The wider inter-mode frequency gap in the tapered wire arises from the spatially non-uniform current density as well. Since the two bullet modes are spatially separated along the wire length, they are exposed to different spin current densities in the tapered nanowire device. This results in significantly different amplitudes of the two bullet modes, as illustrated in Fig. 5(e), and due to the nonlinear frequency shift, an enhancement of the inter-mode frequency gap.

The reduced phase noise of the tapered nanowire STO can be also explained by the spatially non-uniform spin current density. In the straight nanowire devices, the bullet mode position is weakly confined to the center of the nanowire by its interaction with the active region boundaries. Therefore, its position along the wire is highly susceptible to thermal fluctuations, resulting in fluctuations of measured voltage signal. In contrast, the bullet position in the tapered nanowire is mainly determined by the nonuniformity of the applied current density. Therefore, there is a current-induced restoring force that reduces the amplitude of thermal fluctuations of the bullet position along the wire length, thereby reducing the mode’s phase noise. Another important effect which reduces the phase noise in the tapered nanowire STO is the enhanced bias current range of a single-mode generation. This results in higher generation power in the single-mode regime, which decreases the phase noise of the generated signal^22^,^56^.

## Discussion

In this work, we demonstrate that phase noise of nanowire-based spin orbit torque oscillators can be significantly reduced via the nanowire shape design. We experimentally show that the single-mode regime of the STO operation is extended over a wider current range in the tapered nanowire STOs compared to the straight nanowire STOs. The degree of spectral purity of the microwave signal generated by the tapered nanowire STO is also significantly improved in comparison to the straight nanowire STO devices.

To understand the observed effect of the nanowire shape on the STO operation, we developed a one-dimensional Ginzburg-Landau model of a nanowire STO. This model reveals that non-linear self-localized bullet modes are excited in the nanowire under the action of spin orbit torque if the excited spin wave modes possess negative non-linear frequency shift. These bullet modes have micrometer-scale spatial dimensions, which is an order of magnitude greater than the dimensions of the bullet modes excited in point contact STO devices, due to the enhanced role of magnetodipolar interaction in one-dimensional systems.

The model demonstrates that spatially non-uniform spin current density in tapered nanowire STOs is the key factor leading to the phase noise reduction in these devices. The non-uniform spin current density results in a current-induced displacement of the bullet mode from the nanowire center towards the region of lower current density, which extends the single-mode generation regime to a wider range of bias currents. In addition, the non-uniform current density provides a restoring force that reduces the amplitude of thermal fluctuations of the bullet mode position along the nanowire length, thereby decreasing the STO phase noise. The model also predicts a transition to a double-mode regime of the STO operation experimentally observed at higher values of the bias current.

## Methods

### Sample fabrication

Fabrication of the STO devices starts with sputter deposition of a 5 nm thick Pt layer onto a c-plane sapphire substrate at 585 °C followed by annealing for 1 hour at the same temperature, which results in the growth of a continuous Pt film, as verified by high-resolution SEM and atomic force microscopy imaging. Then, straight and tapered nanowires are defined on top of the Pt film via e-beam lithography, brief Ar plasma cleaning immediately followed by *in situ* room temperature sputter deposition of AlO_*x*_(2 nm)/Py(5 nm)/Pt(2 nm) trilayer and lift-off. The AlO_*x*_(2 nm) capping layer is employed to prevent oxidation of the Py layer. The Au(35 nm)/Cr(7 nm) leads are defined via e-beam lithography and e-beam evaporation of the Au/Cr bilayer followed by lift-off. At the final fabrication step, Ar plasma etching is used to remove the 5 nm thick bottom Pt layer everywhere, but under the Py nanowire and the Au/Cr leads.

The liftoff technique employed for fabrication of the nanowire STOs minimizes the nanowire edge damage, thereby decreasing the critical current for excitation of the edge mode self-oscillations. The critical current for excitation of the edge modes is lower than that for the bulk modes for the samples studied in this work. This result is in contrast with our previous study of nanowire STOs^11^, in which the critical currents of the bulk and edge groups of modes were nearly identical. The reason for this difference is the different methods of the device fabrication. Ar plasma etching of the nanowires employed in ref. 11 creates a significant edge damage, increases the damping parameter in the edge region, thereby increasing the critical current for excitation of the edge modes.

### Micromagnetic simulations

Micromagnetic simulations are performed by using a modified version of the MuMax3 software package^57^. The computational domain containing the entire 6 *μ*m long nanowire is discretized into 4096 × 256 × 1 cells, which results in the cell size of approximately 1.5 × 1.5 × 5 nm^3^. The saturation magnetization *M*_*s*_ = 530 · 10^3^ A/m and the exchange stiffness *A* = 0.5 · 10^−11^ J/m were previously determined for this type of STO samples^11^. Spin wave eigenmode frequencies of the nanowire are found as peak positions in the Fourier transform of the dynamics magnetization excited by a sinc-shaped out-of-plane magnetic field pulse of 0.5 Oe amplitude and 50 ps duration^58^. The eigenmode spatial profiles are reconstructed by plotting the cell-specific Fourier amplitudes at the mode eigenfrequency. The auto-oscillatory mode of the system is found by solving the LLG equation with anti-damping spin torque applied to the 1.9 *μ*m long active region of the nanowire. The simulation time is set to 2.5 *μ*s to minimize the transient contributions to the self-oscillatory dynamics. The spatial profile of the auto-oscillatory mode is reconstructed by plotting the cell-specific Fourier amplitudes at the self-oscillatory mode frequency.

### Numerical solution of the Ginzburg-Landau equation

The Ginzburg-Landau equation (Eq. (1)) is derived for a dimensionless complex magnetization amplitude *b*, which is related to the dynamic magnetization components as 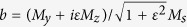. Here *ε* describes the ellipticity of the magnetization precession, which is assumed to be constant. The values of *ω*_0_ and *σ* used in the simulations are chosen to fit the experimentally measured frequency of self-oscillations at the threshold, and the threshold bias current *I*_*c*_. The value of the nonlinear frequency shift *N* = −0.1*ω*_*M*_ is derived from the measured dependence of the first self-oscillatory mode frequency on the bias current^22^. The other material parameters: 
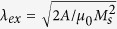
, *ω*_*M*_ = *γμ*_0_*M*_*s*_, *α*_*G*_ are chosen to be identical to those used in our micromagnetic simulations. The integral kernel *G*_*yy*_ is approximated by the Green’s function of a 25 nm wide wire^59^, because the localization length of the edge mode given by our micromagnetic simulations is approximately 25 nm. The spatial domain is discretized into sufficiently small cells, and the resulting set of equations is solved in the time domain starting from a random distribution of *b* until a stationary state of magnetic self-oscillations is reached. To illustrate dynamics of the relative phase between the two bullets in the double-bullet solution of Eq. (1), we multiplied *b*(*t*) by exp[*iω*_1_*t*], where *ω*_1_ is the frequency of the first bullet mode. The relative phase between the two bullets shown in Fig. 5(b,e) is simply 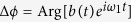.

## Additional Information

**How to cite this article**: Yang, L. *et al.* Reduction of phase noise in nanowire spin orbit torque oscillators. *Sci. Rep.* **5**, 16942; doi: 10.1038/srep16942 (2015).
